# Innate Immunity of Neonates and Infants

**DOI:** 10.3389/fimmu.2018.01759

**Published:** 2018-07-30

**Authors:** Jack C. Yu, Hesam Khodadadi, Aneeq Malik, Brea Davidson, Évila da Silva Lopes Salles, Jatinder Bhatia, Vanessa L. Hale, Babak Baban

**Affiliations:** ^1^Children’s Hospital of Georgia, Medical College of Georgia, Augusta University, Augusta, GA, United States; ^2^Department of Oral Biology, College of Dental Medicine, Augusta University, Augusta, GA, United States; ^3^College of Veterinary Medicine, Ohio State University, Columbus, OH, United States

**Keywords:** innate immunity, innate lymphoid cell, neonate immunity, milk, microbiome

## Abstract

Many important events occur at birth. The fetus is suddenly removed from a protected intra-uterine environment that is aquatic, warm, and nearly sterile, to the dry, cold external world laden with microbes. To survive, the neonate must interact with many organisms, making use of some, while vigorously defending against the others like a nation conducting trade with friendly countries and guarding against hostile ones from invading it, waging wars if necessary. Although, the neonatal immune system is plastic, however, it is highly tolerant which is due to both the fetal development during gestation as well as significant sudden changes in fetal environment and enormous exposure to the new antigens and intestinal bacteria and their products. This “quiescent mode” of innate immune system is part of a highly regulated process to fulfill all requirements of multi-layered process of early life, implemented effectively through the cells of innate immune system. While, most of the neonatal innate immune cells (e.g., neutrophils and monocytes) present contained activity and lower frequencies compared to their adult counterparts, innate lymphoid cells (ILCs), a distinct cellular component of innate immunity, show higher level of activity and presence during period of infancy compared to later stages of life and adulthood, which may suggest a role for ILCs in variable susceptibility to certain conditions during life time. In this review, while we focus on the characteristics and status of ILCs in neonatal immune system, we also draw an analogy from a national defense perspective because of the great similarities between that and the immune system by providing the known biological counterparts of all five core operational elements, the five Ds of defense, detection, discrimination, deployment, destruction, and de-escalation, with special focus on innate immunity, maternal support, and influence during the neonatal and infancy periods.

## Introduction

**Figure 1:**
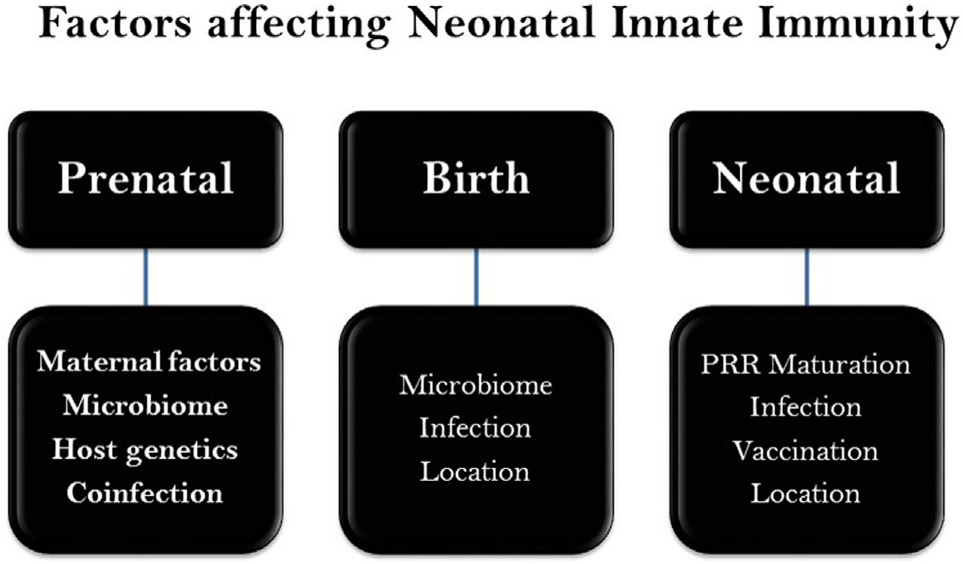
Chronicle scheme of major factors affecting neonatal innate immunity. A wide range of genetic and epigenetic factors may influence the neonatal development during gestation (prenatal) as well as perinatal (post gestation) and neonatal stages. There maybe a level of overlap among factors.

Neonates are dynamic and developing living complex with enhanced vulnerability to a wide spectrum of infectious and non-infectious diseases and conditions (1, 2). This augmented sensitivity requires a fully equipped immune system with prepared alert units to engage at a moment’s notice, and unleash their forces, once proper identification has been made. However, neonatal immune system is a developing structure, evolves in a convoluted step-wise manner (1–4). Infantile immunity is a true elaborate system, simply because while it exits the friendly intra-uterine environment, it is entering the hostile microbe-laden external world. In many ways, the immune system we are born with is the product of the immune environment during pregnancy (1, 4). It is crafted and built block by block and day by day, forged through continuous and never ending improvement during gestation. While still responding to all allo-antigens, the maternal immune system must be tolerant to the fetus, even though it is haplo-mismatched, or semi-allogeneic (half of the antigens being of paternal, and therefore of foreign origin). From the fetal perspective, its immune system must develop the ability to target future pathogens, ones that are completely unknown, while remaining tolerant to self and maternal antigens, as well as some unknown future commensal microbial organisms (Figure 1). This results in immunomodulation during pregnancy and extends to early life. Initially, the tolerogenic status of neonatal immunity was attributed to the immaturity and lack of memory within immune components during infancy. This premise of immune immaturity was replaced by the notion of immunodeviant characteristic of neonatal immunity (1, 5). However, in light of recent discoveries, mounting evidence supports the concept that infantile immunity is in fact a highly regulated, but intellect, orchestrated, functional, and dynamic network of competent molecular and cellular components. Considering the flexibility of neonatal immune system in response to outstretched number of stimulants make it staggeringly simple to further adopt the idea of regarding the infantile immune system as a vigilant establishment rather than immature. This wakeful immune scheme plays pivotal roles in protecting the growing and developing infants from pathologic conditions (e.g., inflammatory situations) as well as providing adequate and appropriate defense against infections by promoting immature or deviant to highly mature responses. Therefore, it is increasingly necessary and crucial to explore, identify, and understand all components and mechanistic pathways responsible for the neonatal immunity in attempt to promote the infantile healthcare and treating newborn diseases.

In this review, we will focus on the innate immunity of the neonates and infants, with particular emphasis on the class of tissue-resident non-BCR and non-TCR expressing lymphoid cells discovered in 2010, the innate lymphoid cells (ILCs). ILCs nomenclature is analogous to that of T cells, divided into two groups of cytotoxic and a helper-like cell. Those comparable to cytotoxic T cells are represented by natural killer cells (NK cells) and helper ones are typified by three subgroups of ILCs, ILC1s, ILC2s, and ILC3s, based on their cytokine secretion and transcription factor profiles. It is noteworthy that ILCs appear and are programmed at embryonic stages of life (6–9). Therefore, it is plausible to suggest that while infantile immune system is under development during perinatal stage, ILCs are playing an important role in protecting the neonate by providing the mechanistic and functional tools to respond rapidly to the transition from a sterile intrauterine environment to the complex nature of external world and differentiate what is to be tolerated from microbes that need elimination through vigorous host responses. Further through this review, we continue with an inventory of the problems faced by neonates and infants and propose theoretical solutions based on military doctrines. The objectives are to enable a practical and deep understanding of neonatal innate immunity at a mechanistic level and glimpsing into immune ontogeny, by drawing parallelism with warfare when appropriate.

## The Status of Neonatal Immune System

**Figure 2:**
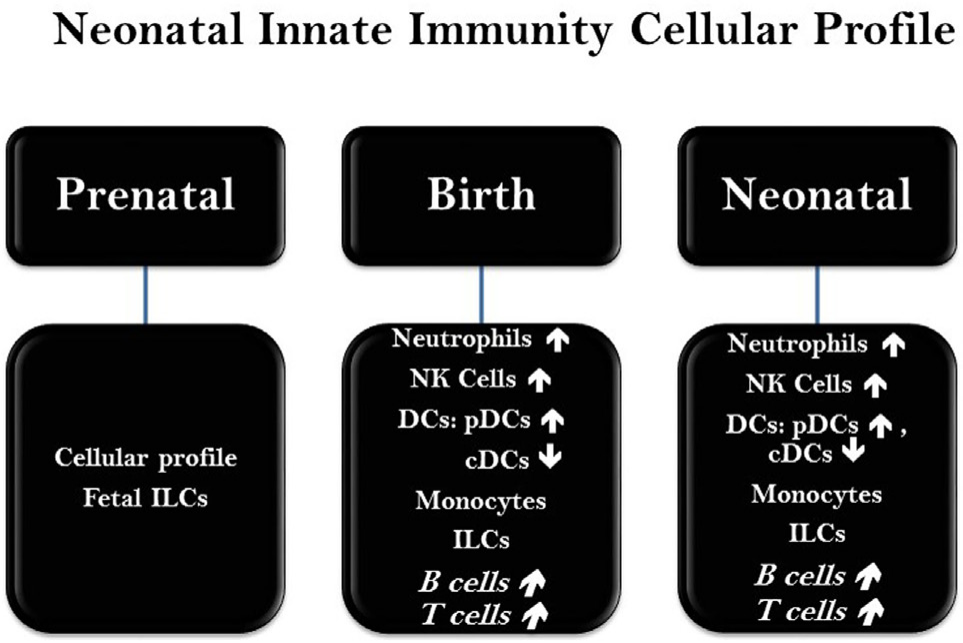
Status of major immune cells at neonatal phase. The presence, frequency, and function of immune cells in neonates are varied in a chronicle fashion and based on the genetic and epigenetic factors at early stages of life. Abbreviations: NK cells, natural killer cells; DCs, dendritic cells; cDC, conventional dendritic cells; pDC, plasmacytoid dendritic cells; ILCs, innate lymphoid cells.

The innate immune responses are the first line of host defense. Although both embryo and neonate confront a complex set of immunologic conditions, however, each phase has its own specific distinct requirements (1, 3, 10). While fetal stage necessitates heightened counter-inflammatory responses against any reaction to the hosting mother, establishing a robust immunologic balance during the transition from a sterile intra-uterine environment to a hostile and diverse world of foreign antigens is highly crucial for the survival of newborn (3, 4). That is why neonatal immunity is a “vigilance complex system” rather than a silent, bystander, and immature arrangement, which possess the capability of eliciting a rapid but smart and selective response to different conditions. This immunodiversity in response is well characterized by functional down-regulation of neonatal leukocytes (e.g., neutrophils, monocytes, and NK cells). Due to certain placental mediators (e.g., progesterone and prostaglandins), Th2 type responses are promoted during fetal stages, which extends through perinatal phase (1, 3–5). In fact, several reports suggest that this pre-determined Th2 propensity from embryonic phase maybe responsible for the absence of inflammatory functions of infantile leukocytes, leading to immunotolerance and low control over infections. At the cellular level (Figure 2), although, frequencies of neutrophils increase transiently just before birth, however, neonatal neutrophils are presented by lower number and subdued quality and function. Neonatal neutrophils are not able to form neutrophil extracellular trap (NETs) which affects their capability of killing bacteria effectively. Also, neonatal neutrophils have decreased expression level of adhesion molecules (e.g., L-Selectin: CD62L, Integrin: MAC-1), affecting their binding to endothelium. Lower expression of TLRs including TLR2 and TLR4 as well as decreased level of certain pivotal immunophysiologic features of neutrophils including, not limited to, phagocytosis, neutrophilic burst, and diminished capacity of degrading intracellular infective agents are all features of neonatal neutrophils. Same scenario of neutrophils is applicable to the neonatal antigen-presenting cells (APCs). Basically, neonatal APCs including macrophages and dendritic cells (monocytes) show decreased expression of MHC-II which may result in imperfect function and inactivity. Further, neonatal APCs possess lower expression of co-stimulatory molecules (e.g., CD80/CD86) as well as TLRs (1–5). Collectively, neonatal monocytes (DCs and macrophages) show a tendency toward a Th2 type response demonstrated by decrease in MHC-II, low co-stimulatory/adhesion molecules, higher ratio of plasmacytoid DCs to conventional DCs, lower chemotaxis, reduced extravasation, and migration along the endothelium, ensued by diminished inflammatory responses which may make neonates more vulnerable to infective agents.

Complement system is another important component of innate immunity. Neonatal complement system is under development and does not function in its full capacity. All major components of complement cascade, such as C1q, C4, C3, properdin, and factor B are decreased in newborns which may lead to higher susceptibility to the infections and other pathological conditions (1, 3).

Natural killer cells are a distinct lineage of lymphoid cells, lacking CD3, but expressing CD56 and NKp46 are part of innate immune system against viral infections and tumors. During early stages of gestation, NK cells are highly regulated and very hypo-responsive to target cells as part of their protective role as fetus is developing (11, 12). Later, throughout the gestation, NK cytolytic function increases compared with earlier phases. At birth NK cells are presented in high frequencies, but lower toxicity. However, they show a reduced level of threshold for activation which renders anti-viral protection. Throughout the neonatal stages and even during the next few days after birth, NK frequencies decreases and reaching adult levels by 5 years of age (3, 12). Importantly, it is demonstrated that expression of NK cells’ receptors plays a pivotal role in host susceptibility to autoimmune and inflammatory diseases. It is documented that expression of receptors in NK cells (e.g., LY49 and NKG2A) is highly regulated during ontogeny and whole lifetime (13). Both human and animal studies have indicated that the balance between stimulatory and inhibitory receptors plays a central role in susceptibility, function, interaction, and activation of of NK cells (13, 14).

Most importantly, under a new classification, NK cells are categorized as ILCs, including ILC1, ILC2, ILC3, and the lymphoid tissue inducer (LTi) cells. While, both NK cells and ILC1s are producing interferon-γ (IFN-γ) and tumor necrosis factor (TNF), however, NK cells are differentiated from ILC1s and all other ILCs by possessing cytolytic function. In addition, NK cells express a variety of activating and inhibitory receptors, including NKG2D, Ly49 or KIR, CD94–NKG2 heterodimers, and natural cytotoxicity receptors, as well as co-stimulatory receptors (1, 3, 13).

Given their role in sowing of intestinal tissues and lymphoid system throughout the embryonic stages, it is clear that ILCs play fundamental functions during embryogenesis. Their pivotal roles are more extended further after birth and through neonatal phase, particularly considering their capability of rapidly responding to environmental signals, containing infections, and maintaining tissue homeostasis. All these provides strong rationale not only to explore more about ILCs but also test the potential of targeting ILCs as therapeutic modality in the treatment of neonatal diseases.

## Innate Lymphoid Cells

Innate lymphoid cells, characterized and reported in three important 2010 publications, belong to a new class of lineage-negative (Lin-) lymphoid cells that mediate both pro-inflammatory and anti-inflammatory responses (6, 7, 15–17). Though Lin- (Lineage negative), they express CD25, CD127, IL-2 R-β, and IL-7 R-α. Rather than specific antigens, they target conserved, shared components of the pathogens, and thus do not require recombination or expansion from memory cells. The presence of ILCs in different tissues throughout the body is specified. They are implicated in the development of tissue microenvironment, structure, composition, and recovery from very early embryonic stages through the whole lifetime (7, 18–20). Like true sentinels, ILCs respond with rapidity, measured in minutes to hours. Many ILCs do not possess cytotoxicity; rather, they release highly bioactive mediators in an auto- and paracrine manner. Based on their transcriptome and cytokine profiles, there are five subtypes of ILCs. ILC1 expresses transcription factor T-bet and releases TNFα and IFNγ when stimulated. Based on the CD127 level, there are two subtypes of group 1 ILC: those that are CD127−, which produces both TNFα, and IFNγ when stimulated by IL-12, IL-18, and IL-15, and those that are CD127+, which produces only IFN-γ in response to IL-12 and IL-18 but not IL-15. NK cells are very similar to CD127− ILC1 in cytokine profiles with the exception that they possess cytotoxicity and produce perforin and granzyme. ILC2 expresses GATA-3 and produces type 2 cytokines: IL-4, IL-5, IL-9, IL-13, and amphiregulin, a homolog of EGF. ILC2 is similar to Th2 in that it can provide T-cell-independent B-cell help and is important in wound healing and tissue homeostasis. Because of these functions, group 2 ILCs are particularly adapted in defense against helminthic infections. ILC3 expresses RORγt and produces IL-17A and GM-CSF, if CCR6+. This subtype of ILC3 has lymphoid tissue inducing, LTi, ability. If CCR−, ILC3 can differentiate further to express natural cytotoxicity receptor (NCR) and produce IL-22 in addition to TNFα, GM-CSF, and IFNγ. Because of the ability to make IL-17 and IL-22, group 3 ILCs are something known as ILC17 and ILC22, respectively.

Since the neonatal gut is sterile and must cultivate its own microbial flora, ILCs are key to the co-evolution of the intestinal microbiome and adaptive immunity of the infant (7). At the intersection of innate and adaptive immune systems, ILCs occupy a central role in coordinating inflammation, immunity, wound healing, and tissue homeostasis, with a wide range of influence from metabolism to tumor defense to obesity (21). They accomplish these varied tasks through intimate interactions with macrophage and classic adaptive immune cells: T- and B-cells. Based on the functional demands and the capabilities, maternal ILCs are ideally suited to assist the neonate. Supporting this conjecture, a report documenting ILCs in human milk appeared very recently (22). We have investigated the presence of ILCs in human infant oral epithelium, and have found their presence with a predominance of ILC2 (23). These cells are GTAT-3+ and produces similar interleukins to type 2 T helper cells, known to be important in the defense against extracellular bacterial pathogens. ILC2s are also reported to contribute to the development of asthma during neonatal stages in mice by expression of IL-13 throughout the early-life viral infection (e.g., rhinovirus), causing mucus hyper-secretion and promoting airways responsiveness. Therefore, it is plausible to suggest that ILC2s maybe considered as a therapeutic target in the treatment of neonatal respiratory disorders such as asthma (24, 25). Further, ILC2s are reported to promote asthma at very early age of life through an IL-33-dependent mechanism. This is mainly attributed to the accumulation of ILC2s as well as mast cells, basophils, and eosinophils in the developing lungs during both perinatal and postnatal period. Therefore, it is plausible to explore the potential of IL-33 axis as an immunotherapeutic target in the initiation, progression, and treatment of asthma (26–28).

It is also reported that neonatal ILCs, most probably ILC3s may prevent lymphopenia-induced proliferation (LIP) (29). LIP is a condition associated with T cell activation, memory differentiation, tissue destruction, and a loss of TCR diversity. It is strongly suggested that T cell homeostasis in neonatal mice is regulated by mechanisms that are fundamentally different to adults. Accordingly, it is shown that IL-7R-dependent ILCs block LIP of CD8+ T cells in neonatal but not adult mice. This ILC-based inhibition of LIP ensures the generation of a diverse naive T cell pool in lymphopenic neonates that is mandatory for the maintenance of T cell homeostasis and immunological self-tolerance later in life. Moreover, this make it plausible to test the hypothesis that neonatal ILCs might lose their suppressive function in an age-dependent manner, remains to be more comprehensively interrogated.

**Figure 3:**
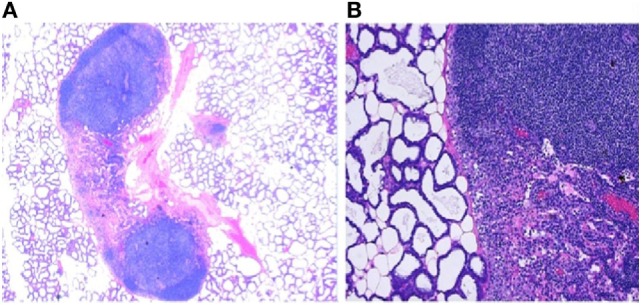
The presence of immune components in mammary glands. **(A)** Low power photomicrograph of lactating murine (C57BL/6) mammary gland showing lymphoid aggregates juxtaposed to the alveoli. These secondary lymphoid tissues (SLT) are the “base camps” for lymphocytes where further differentiation and acquisition of functionality such as cytokine production and cytotoxicity occur. The strategic location of SLT to the milk glands allows lymphocytes to defend the lactating breast as well as deployment to the infant through milk. **(B)** High magnification of the histology of lactating mouse mammary tissue, H and E stain, showing afferent and efferent lymphatic vessels, capillaries, and follicular formation with densely packed heterogeneous small lymphocytes in close proximity to active secretary units made up of cuboidal alveolar lactocytes. This is not different from the aerial photograph of a military base with tens of thousands recruits undergoing training where inbound and outbound roads bring in supplies and remove wastes.

Further, a novel role for ILC3s, through an IL-23-mediated mechanism has been reported which may attribute a biotherapeutic role to ILC3s in the treatment of neonatal intestinal inflammation. Accordingly, any intervention (e.g., antibiotic therapy) may cause alteration of commensal flora, increasing penetration of bacteria or bacterial products through areas of ILC3-induced increased permeability located in the anlagen (30). This condition may lead to uncontrolled bacterial proliferation, cytokine storm, and death. This is specifically important because ILCs neither possess nor need rearranged-specific antigen receptors like regular lymphocytes, their response can be more rapid, usually within minutes. This sentry function is further enhanced by the fact that they reside near borders where heavy microbial contacts occur, such as skin and the lining of aero-digestive tracts (Figure 3). The mucosal and cutaneous barriers encounter both pathogenic and commensal microbes. It is possible that maternal ILCs present in the milk serve four important functions for the infants: they defend against pathogenic microbes, shape the oral and intestinal microbiomes, maintain the integrity of the immature mucosal borders, and modulate the maturation of the neonatal immune system. In addition, from the maternal perspective, lactating mothers must guard against microbial infection of the breasts—one-third of the premature cessation of breastfeeding is due to mastitis. Milk leukocytes may well perform such a protective function (31). These leukocytes are haplo-mismatched with infant’s MHC. How they escape rejection and how the babies avoid graft-versus-host attacks, with cytotoxic T-cells making up 3% of total milk leukocytes, are most interesting and largely unknown. In addition, it is clinically important to understand how milk shapes the intestinal microbiome of the infants, as well as the development of their T and B cell repertoires. Altogether and whether ILCs involve other innate leukocytes (e.g., neutrophils) remains open to further investigations.

## The Transfer of Mediators and Cells by Milk: ILC’s Status in Human Milk

Neonatologists and immunologists now regard milk as a dynamic living fluid, capable of modifications to meet the changing demand of the infant (32). Human milk contains at least 32 non-cellular bioactive elements from carbohydrates to lipids, to proteins, and 5 cell types (33). The presence of cells in fresh, unpasteurized human, and animal milk have been characterized since the late 1960s; they include neutrophils, macrophages, lymphocytes, stem cells, epithelial cells, and microbe. A newborn ingests an estimated 10^8^ maternal cells per day with 80% being macrophages, originating from maternal peripheral blood monocytes, probably of gastrointestinal origin (34). More than 90% of these cells are viable. With local or systemic infections in either the mother or the baby, this maternal cellular transfer increases rapidly, exceeding 10^9^ cells/day. These bioactive molecular and cellular milk components protect the breast from infection while modulating the development and maturation of the neonatal immune system (35). Among the non-cellular factors in the milk, many have antimicrobial properties. For example, lactoferrin B, formed from digestion of lactoferrin, is a potent agent against both Gram-positive and Gram-negative bacteria. That lysozymes and lactalbumin share similar amino acid sequences further supports the concept that milk is an antibiotic (36).

Together, these cellular and biochemical mediators cultivate and shape the developing gut microbiome of the infant. The luminal mucous layer has a particularly important reciprocal relationship, affecting and affected by, the epithelial barrier and the luminal microbiota (33). Breast milk thus contains both prebiotics (oligosaccharides) and microbes that are critical in colonizing the infant gut as well as helping infants in the digestion of the nutrient components of milk.

**Figure 4:**
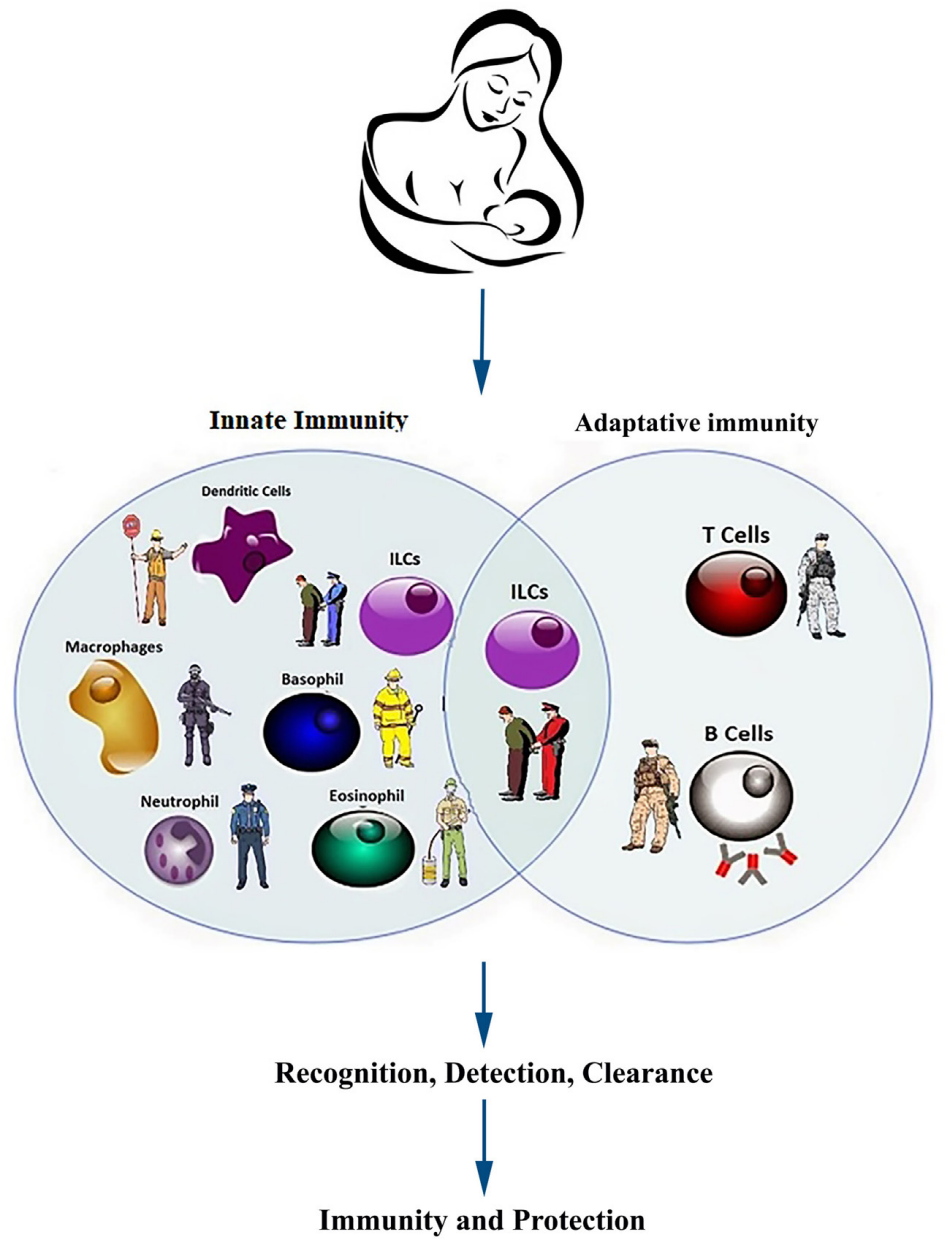
Components of innate and acquired immunity in infants and neonates. Heuristically, the immune system defends the neonate and infant against infections in similar manners as the military and the police keeping the country safe. The young immune system must develop *de novo* to respond to some foreign antigens (pathogens) and more rarely, abnormal autoantigens (neoplastic and auto-reactive cells), while accepting some other foreign antigens (commensal microbes) and normal autoantigens. Multiple components from innate and adoptive immune system collaborate to accomplish the above tasks with multiple redundancies to ensure robustness. The active defense against pathogens, like many joint operations in the military, requires coordination of multiple units from different branches of the armed forces. Innate immunity is faster to respond (seconds to minutes) but the duration of action is shorter (3–5 days). In contrast, the adaptive immunity takes longer (4–7 days) to activate but the response is much more specific and sustained (weeks to years).

Innate lymphoid cells occupy the key intersection between adaptive and innate immunity, functioning like elite foreign troops sent into vulnerable regions to boost and train defenses, establishing a safe and sustainable local environment (Figure 4). ILCs of human milk may shape the infant or a land intestinal microbiomes by modulating neonatal immunity. How maternal ILCs modulate the infant ILC populations also remains largely unknown. The immature immune system of the newborn must rapidly respond to the transition from a sterile intra-uterine environment to a microbe-laden external world and differentiate what is to be tolerated from microbes that need elimination through vigorous host responses. Furthermore, lactating mothers must guard against microbial infection of the breasts; the milk leukocytes provide such defense (22). Milk ILCs may impart innate immunity in newborns. The next step is to investigate how they shape neonatal immunity and microbiome. The oral transfer of maternal cells through milk clearly occurs, and these cells (e.g., ILCs and other leukocytes) survive the gastric pH to live in the intestine of the neonate for 6 days (22). Although documented, however, how mammary glands sense and respond to the changes in the infant microbiome is largely remained unclear for now.

## Neonatal Gut Microbiome and Immune System

Primary lymphoid aggregates form *in utero*, deep to the crypts of Lieberkühn. These initial cells of small bowel mucosa-associated lymphoid tissues express VCAM-1 and are CD127+/CD4+. They belong to the LTi, and share similarity with ROR-γt+ ILC3. Responding to lymphopoietin released from the developing thymus, LTi cells form organizing centers followed soon by the appearance of CXCL13+ fibroblast stromal cells and CD11c+ cells. At birth, perhaps due to products of transcription factor ROR-γt, CD4+, and CD8+ T cells predominate Peyer’s patches, with CD8+ cells out numbering CD4+ cells by 4 to 1, though both lack the experience of antigenic challenges. Maternal milk leukocytes, 80% myeloid and 20% lymphoid, a ratio similar to peripheral blood leukocytes, contribute to the newborn’s mucosal lymphoid tissues. The origin of milk lymphocytes, interestingly, is maternal Payer’s patches. In infant Peyer’s patches, most cells of maternal origin are CD8+ lymphocytes and they express CCR9 and α4β7 integrin, known for intestine-homing. Compared to the relatively naïve infant CD8+ T lymphocytes, phorbol ester, and ionomycin stimulation of maternal origin CD8+ T cells produced two- to four-fold more TNFα, IFNγ, and IL-18 (37).

At birth, the neonatal gut suddenly has two new tasks: nutritive and defensive. The former involves digesting and absorbing nutrients, and the latter involves the five Ds with every microbial encounter, both are critical to survival. Maternal intestinal microbiome contains 450 species while infants have far fewer. Seventy-two percent of the 187-specific operational taxonomic units (OTU) found in intestinal microbiome of vaginally delivered neonates are identical to maternal OTUs. In the absence of “trained and experienced” T and B cells, with a weak mucosal barrier, and a lumen minimally populated by microbes, the neonatal immune system must differentiate commensal from pathogenic microbes. The gastrointestinal tract faces an onslaught of pathogens while performing its digestive and absorptive duty (38). For most infants, the gut carries out these functions efficiently and safely. About 5% of the time, especially when birth weight is low, nominal operations fail and necrotizing enterocolitis (NEC), a potentially fatal condition, develops (39). Without mother’s milk, the formula fed infants have a much higher mortality rate compared to those that received breast milk: for every 100 mL/kg increase in breast milk intake during the first 2 weeks of life, there is a reduction in mortality risk by 0.87 (40). The more than 200 unique milk glycans capable of binding specific microbes, and the high levels of lactoferrin, contributes to this great reduction in mortality. Some of the intestinal injury in NEC is due to inflammation, like cities destroyed by war. The high TGFβ level and IL-10, may impart check and balance by suppressing inflammation while also providing transient tolerance of maternal lymphocytes to the infant host and the infant host to maternal cells.

## Gut Microbiome Acquisition

Many factors shape an infant’s gut microbiome, ranging from host genetics (41) to weaning diet (42). The earliest exposure to maternal microbes occurs *in utero* (38). Microbes have been detected in amniotic fluid, the umbilical cord, the placenta, and the meconium of healthy term babies (43–46). In one study examining gut microbiome acquisition, pregnant mice were orally inoculated with a genetically labeled microbe. Pups from these dams were obtained by cesarean section 1 day before predicted labor, and the labeled strain was isolated and identified in the meconium of these pups indicating vertical transmission of gut microbes *in utero* (47). The birthing process is the infant’s next and critical exposure to microbes. Delivery mode greatly impacts infant gut microbial composition: infants born vaginally have a gut microbial community that more closely resembles the maternal vaginal microbial community, while infants born *via* Cesarean section have a gut microbial community that more closely resembles skin flora (48). Infant diet immediately after birth (breast milk, formula) represents the next major factor that shapes the infant gut microbiome. Breast milk, in addition to containing maternal immune cells and cytokines, also contains oligosaccharides and the microbes necessary to metabolize these oligosaccharides—an optimal inoculum for healthy infant gut development (32, 49, 50). Clinical studies comparing maternal areolar skin and milk flora to the infant gut microbiome in 107 healthy maternal-infant dyads showed definitively the seeding of the neonatal gut by the maternal sources. During the first month of life, milk and areolar skin flora accounted for 27.7 and 10.4%, respectively, of the neonatal gut microbiome. Each mother has a unique areolar and milk flora, and these microbial communities correlate with her baby’s gut microbiome in a dose-dependent manner. Metagenomic function analyses predicted that maternal flora have significant influence on carbohydrate, amino acid, and energy metabolism. Such maternal influence continued even after the introduction of solid foods (32).

These early exposures to microbes can have long-term effects on infant health and immune development. Gnotobiotic mice, also known as germ-free mice, demonstrate abnormal immune development including underdeveloped Peyer’s patches and mesenteric lymph nodes, defects in antibody production, and impaired maturation of lymphoid follicles (51). These defects indicate that gut microbiota play a key role in normal immune development. Infant delivery mode and the associated microbial communities acquired with each mode have also been linked to long-term health effects including increased risk of immune-mediated diseases in infants born by Cesarean delivery (52). This indicates that both microbial presence and microbial composition are essential to healthy immune development in infants.

Specific strains and combinations of microbes have also been linked to increased host production of anti-inflammatory cytokines (IL-10) and increased abundances of TGFβ-expressing T cells—again demonstrating that critical interactions occur between gut microbes and the host immune system (53).

The cross-talk between ILCs and microbiota demonstrates the significance of ILCs’ role not only in establishing and maintaining homeostasis but also in early resistance to infections. The Promyelocytic Leukemia Zinc Finger (PLZF) has been suggested as one of the mechanistic transcription factor by which ILCs are enabled to both respond to commensals and regulate the composition and/or quantity of the microbiota (54). Using this cellular/molecular approach, it can be envisioned to test the possibility of modulation of PLZF in human hematopoietic cells (e.g., in an *ex vivo* model or in humanized mice) as a translational therapeutic method in re-establishing and maintaining homeostasis.

## Innate Immunity, an Army Inside: An Analogous to Military Defense System

Neonates are dynamic and as the late Charles A. Janeway, Jr. correctly postulated almost three decades ago, the effective detection of microbial antigens must possess three essential characteristics. First, the invaders have some antigens that are different from host antigens, making it possible to differentiate them from self. Second, there is conservation among some of these non-host antigens so that specific individual identification is not necessary, reducing the magnitude of the surveillance required, and improving the efficiency of detection. Third, these conserved, common antigens are essential for the invaders, such that they cannot mutate and do away with them (55). From this evolved the concept of pattern recognition molecules and pathogen associated molecular pattern (PAMP). The receptors that bind pathogen-associated antigens have expanded greatly and now include eight major classes divided into three functional groups. TLR, NLR, and RLR make up the first group; they mediate inflammation and apoptosis. The TLRs, or TOLL-like receptors, are evolutionarily conserved receptors, some on the cell surface (TLR1, 2, 4, and 6) others are endolysosomally associated (TLR3, 7, 8, and 9), which when engaged by the specific target parts of the invaders, illicit rapid and escalating innate immune defense against the hostile agents. The other two members of this group are nucleotide-binding oligomerization domain-like receptors (NLR) and RIG-I-like receptors (RLR). The second group of pattern recognition receptors includes pentraxin, collectin, and ficolin. They mediate opsonization and complement activation. C-type lectin and scavenger receptors make the third group and they mediate phagocytosis. Together, these receptors form an intelligence surveillance network, constantly patrolling the integuments and internal domain for dangerous invaders. Once they detect microbial presence, there are multiple feed-forward cycles which up-regulate the inflammatory and immune responses. The initial, rapid, and responses are the responsibility of the innate immune system. A more specific, sustained, but slower response is from the adaptive immunity.

The innate immune defense are the prepared alert units, fully equipped to engage at a moment’s notice, and unleash their forces, once proper identification has been made through these pattern recognition receptors and command to attack is issued (the signals downstream of TLR binding are MyD 88 and TRIF). There are 10 TLRs in humans and 11 in mice. The rapidity of the response comes, in part, from the readymade, stably coded nature of TLRs, without the need for any somatic recombination of genes, nor transcription or translation to produce novel receptors. The adaptive immunity requires education of the T and B cells by prior antigenic exposure and formation of memory cells. Though slower to respond initially, these traditional lymphocytes have specific receptors through hypervariable recombination and, once expanded produce extremely effective antibodies and cytotoxic T cells.

**Figure 5:**
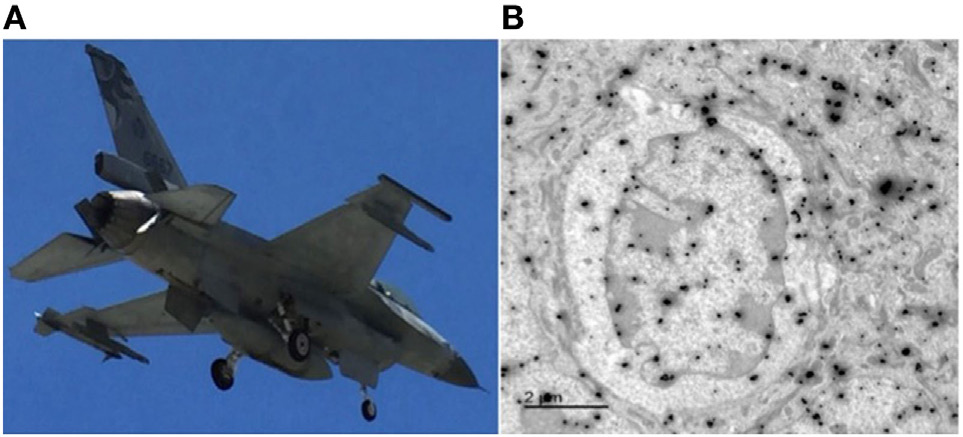
Innate immunity, the army inside. **(A)** A jet fighter must be able to detect any approaching air or sea craft, discriminate friend or foe, and deploy weapon systems that can destroy the foe, and then stand down. Mistaken friendly for foe will cause casualties to one’s own forces; however, failure to detect or discriminate a foe will result in destruction of the unit by the invader. The infrared-guided AIM-9 sidewinder missile carried in the port wingtip launcher is similar to an antibody: released at a distance and capable of target tracking and destruction (Photograph taken by Marian R. Lambert-Yu, 2016). **(B)** Transmission immune-electron microscopy of an innate lymphoid cell (ILC) with two sizes of gold particles labeling the cytokine profiles. This is an important cell, which can sense pathogen presence in its microenvironment and releases appropriate cytokines in response to the invader. Like the fighter jet, innate immune cells, such as macrophage, neutrophils, and NK cells faces the same demands and carries out the same tasks of detection, discrimination, deployment, destruction, and de-escalation. The immune-gold spheres label this ILC’s “payloads” and they many include tumor necrosis factor α, INFγ, IL-5, IL-13, IL-17, and IL-22, comparable to the sidewinder and sparrow missiles carried by the F-16. The difference is that ILC has no “pilot,” it makes its weapons, and its behavior is completely rule-based.

The defense by an organism against the ever-present threat of microbial invasions is exactly like national defense against hostile invaders. There are five Ds that describe the stages of defense by the military across scales from a single soldier, or a fighter jet to a division or a carrier battle group: detection, discrimination, deployment, destruction, and de-escalation (Figure 5). These five Ds are the same tasks faced and carried out by any immune system. Each of the five stages has elaborate, complex, and highly effective biological equivalents. These tasks are considerably more complicated during pregnancy. It has to, often for the first time, use the five Ds to defend itself without autoimmunity. Compounding the difficulty, not only are all five Ds not fully developed, the precise timing of parturition is unknown. Neonates have ineffective antigen presentation. They do not have the same robust response when TLRs bind the cognizant microbial components. Their adaptive immunity has not had the time to build up the necessary memories and repertoires to permit the discrimination and specific immunity. With decreased antigen presentation, the co-stimulation is weak due to low CD28 levels. The frontline myeloid cellular defenses deployed by adults, the neutrophils, are not as easy to recruit and those that are present at the location of microbial invasion are not as capable in phagocytosis and intracellular microbial killing. Perhaps, the only D that is “good” in neonates is the de-escalation: the suppression of inflammation through asymmetrical polarization with low Th1 to Th2 ratio and high FoxP3 to Th17 ratios. The neonatal IL-10 level is ten- to thirty-fold that of adults, as is the level of immune-modulating TGFβ.

Part of the reason for such a suppressed immune state is to assure that, during the critical developmental period of acquiring and building up adaptive immunity, self-reactivity does not happen just as one citizen does not attack other citizens and armed forces of national defense should not attack their own civilians. When a rogue army unit launches attacks on its own citizens, the central government will and must go to great distance to assure that the country has sufficient policy, procedure, and capability to quickly suppress, curtail, or better, prevent that. Such self-tolerance is largely a result of thymic education, where self-reactive T-lymphocytes undergo apoptosis in an MHC-restricted manner. Innate immunity must also possess specificity, but by very different mechanisms. There is no thymic selection for innate immune cells. During the normal tissue turnover, dead cells release their nucleic acids and other intracellular contents. Macrophages engulf this debris, keeping it in their endosomes as they would if it were microbial in origin. TNFα production increases only when bacterial nucleic acids bind TLR 9. How macrophages achieve this discrimination remains unclear, but it is certainly context-dependent and involves transcriptional control of TLR 9.

Faced with an immature adaptive defense compounded by active suppression, the neonatal defense rely more on innate immunity. This sequential appearance with adaptive immunity following innate immunity is another example of ontogeny recapitulating phylogeny—relative to adaptive immunity, innate immunity has been present since the very early eukaryotes (56).

## Scope of the Problems and Potential Strategies

The biotic process is the ultimate warfare for survival. In warfare, at any scale, opposing forces strive to exploit asymmetries, magnifying their own strategic, operational, and tactical advantages while minimizing their weakness. Asymmetries and trade-offs are inevitable and dynamic: opportunities can become liabilities and constraints can turn into strengths. Some asymmetries are extreme and extremely important for the survival of the neonate. Here, we focus on five key asymmetries: size, number, replication time, adaptability, and resource consumption. The neonate has size and mass in the range of 10^−1^ m, and 10^0.5^ kg while the length and mass of bacteria are 10^−6^ m and 10^−13^ kg. Quantitatively, there is only one neonate, but the number of bacteria is in the range of 10^12^–10^15^. Logistics for supplying a neonate, which consumes energy at a basal rate of 10^2^ W, or 10^2^ J/s, is logs of order of magnitude higher than bacteria, which consumes energy at 10^−12^ W per bacterium (57). The neonate has an efficient distributive fractal network—the circulatory system—to supply resources and eliminate wastes to maintain homeostasis of its internal environment. It also has reserves for energy and materials in the form of hepatic and muscle glycogen and adipose tissue. At the level of the individual cells, autophagy and mitophagy provide critical reduction in metabolic rate and resource needs through recycling of the intracellular organelles. Because of their small size, it takes only 30 min for bacteria to duplicate. Under ideal conditions, in 1 day, a single *E. coli*, can expand into 2^48^, or 281 trillion (2.81 × 10^14^). Bacteria are also very adaptive, capable of switching nutrient requirement, and acquiring resistance to antibiotics and other toxins by horizontal plasmid transfer. Their virulence increases drastically as their numbers increase beyond certain threshold—quantity has a quality of its own, a well-accepted military philosophy (58), and a proven microbiological phenomenon (59). The neonate cannot duplicate. It reproduces with a generation time measured in decades, 240,000 times longer than that for the bacteria. Given all these asymmetries, the bacteria, together with other microbes, have great advantages over the neonate in this perpetual war for survival. It is thus not too surprising that even in 2016, according to UNICEF data, 2.6 million babies die in the first 28 days of life (60). The neonatal immune defense can destroy millions and billions of invading microbes but fresh ones will continue to replace the loss. There is only a single neonate comprised of 10^13^ cells, and of course, it has no replacement. Of these 10 trillion cells, some have the responsibility and ability to carry out the defense against microbial invasions. Among these “professional” defense cells, some are garrisoned near the mucosal and integumentary borders as various local tissue lymph aggregates while others circulate (Figure 3). Collectively, these local tissue lymph aggregates are the secondary lymphoid tissues, or SLT, and they serve the critical functions of “base camps” for lymphocytes, where residence, differentiation, and maturation take place, similar to housing, education, and certification functions of a military base. As Lin−, CD127+, and CD135+ common lymphoid progenitors progress through the various stages of maturation, their functionality become more defined and their pleuropotential reduced. It is only with the final maturation that immune cells attain full cytotoxicity and ability to produce inflammatory cytokines, such as IFNγ and TNFα, not too different from the weeks to months of classroom and field instructions and practices before a new recruit can carry a loaded M-16. NK cells, for example, go through five stages of maturation starting from Lin−, CD117+ HSC to mature NKG2D+, CD244+CD11b hi Ly49 ± mNK. All told, 45 surface markers orchestrate this process, with increasing number of markers during each stage (61). 5 × 10^8^ leukocytes circulate in peripheral blood in an adult, a tenth of that is present in peripheral blood of an infant. Myeloid and lymphoid cells make up the circulating leukocytes with a ratio of 3:1. These cells, their products, and maternal transfers make up the neonatal defense (Figure 4).

To exploit the asymmetries for survival, the neonatal defense must manipulate the microenvironment to its advantage. There are many such exploitations; we will describe some common examples. Since the neonate has material and energy reserve, and bacteria do not, depriving them of essential nutrients and trace elements such as transitional metal ions is a sound strategy. Zinc concentration in bacteria, for example, is extremely critical and with a very narrow range of acceptability. Outside this range, the bacteria cannot survive (62). Binding ions of transitional metals, such as Fe, Mg, Ca, Cu, and Zn by chelating proteins such as lactoferrin and other transferrins are an effective method deployed by the neonate. Lactoferrin in particular is of great interest and importance. It has very high affinity for Fe, 300-folds that of transferrin, and even higher in low pH condition associated with infections. The concentration of lactoferrin in colostrum is extremely high, averaging 7 g/L, compared to serum concentration of 0.4–2 mg/L (63). Infants can tolerate higher temperatures than that which is optimal for microbes. Fever is thus a universally deployed defense against infection, usually in response to pro-inflammatory cytokines when the innate immune cells encounter pathogens: IL-6, TNF-alpha, and IFN-gamma. However, due to the low IFN-gamma level, the febrile response in the very early neonatal period may not be as marked (64). Bacteria, even aerobes, have limited antioxidant capacity, while the neonate with an elaborate antioxidant system made up of mitochondria, ascorbic acid, vitamin E, and glutathione, among others, can generate and tolerate an oxidative burst producing hydrogen peroxide, nitric oxide, and peroxynitrites. These highly oxidative agents are very toxic to the microbes. Neutrophils and macrophages kill by precisely such mechanisms often through NADPH-dependent respiratory bursts (65).

At its core, rates determine the outcome of war. The goal is to destroy the opponent faster than it can rebuild. Destroying microbes, which employ mass production, requires mass destruction. Altering the environment to move key variables, such as temperature, oxygen partial pressure, or transitional metal ion concentration, to list just a few, outside the survivable ranges for the bacteria but tolerable to the host can increase the rate of microbial elimination above that of their reproduction. That is the essence of host defense by manipulating the microenvironment. Built into this strategy is the discrimination step of the five Ds, which permits preservation of self and killing of the non-self. Other than environmental discrimination, the differentiation must occur at the cellular level. This discrimination process is complex and critical. Failure at this stage causes autoimmunity due to loss of self-tolerance or fulminant infection due to insufficient antimicrobial response. The maternal transfers of cells and materials, as well as microbes after birth greatly assist the neonate.

## Some Clinical Correlations in Health and Disease

The initial development of the immune system during the neonatal period and the acquisition of the inaugural gut microbiome have significant and long-lasting impacts in the individual’s future health and disease, affecting cardiovascular, metabolic, neoplastic, and neurodevelopmental processes. Many diseases, such as hypertension, atherosclerosis, type II diabetes, obesity, dyslipidemia, celiac disease, psoriasis, autism spectrum disorders, schizophrenia, and Parkinson’s disease, to name a few, are linked to abnormalities in innate immunity and microbiome. There have been many intense investigations attempting to elucidate the precise mechanisms (66). Even though we understand that underpinning many of these pathological states is chronic inflammation, the cause-effect relationship governing the disease process remains unclear. This is because most biological structures and processes are complex adaptive systems (CAS); chronic inflammation is certainly no exception. Every CAS has many loosely connected interacting parts, forming self-organized hierarchical systems (67). Many sub-systems together comprise the supra-system and many supra-systems together form the supra–supra system, and so on up the scale. Each sub-system has many sub-sub-systems and so on down the scale. There are two defining characteristics for CAS. The first is extreme sensitive dependence on initial conditions. The second is the emergence of invariant properties across scales (68). Because of these features, CAS generates fractals, behaves chaotically, and does not lend itself to traditional reductive investigations. Innate immunity, gut microbiome, and energy metabolism are three principle interacting sub-systems forming a single CAS. Perturbation in one ripple through the other two in a dissipative manner and the system restores itself back to the original steady state (attractor). Occasionally, however, the disturbance may be long or large enough that the response of the system is feedforward, in a self-magnifying, exponential way, pushing the system out of existing steady state. When this happens, the system undergoes chaotic shift to a new quasi-stable state (new attractor). Sustained increase of dietary fat can cause changes in gut microbiome (increase in bacteroides and firmicutes) and decrease in intestinal epithelial integrity. This leads to TLR ligands such as LPS and CpG translocating from gut lumen into the systemic circulation, which causes release of inflammatory cytokines from peripheral blood innate immune cells. Responding to the first waves of cytokines, more innate immune cells release their own inflammatory cytokines, and the cycle magnifies. The counter measures, such as TGF-β, IL-4, and IL-10 by ILC2 and Th2 suppress the inflammatory response until the system finds a steady state (new attractor). Knockout murine models (IL-10−/−) definitive confirm the above conjecture (69). Emergent properties manifest only when system is intact. That is why *in vitro* experiments do not always agree with *in vivo* investigations. To understand the reaction at the organism level, we often need to use the organism. Population level phenomenon requires study of the population. That said high-level observations, like epidemiological studies, could and often shed light on possible dynamics that govern the behavior of component parts. For example, epidemiological studies dating back to the 1960s have documented the increase in neuropsychological disorders such as autism spectrum disorder and schizophrenia following outbreaks of rubella from less than 1–13 and 20%, respectively (70). Intra-peritoneal injection of polyinosinic polycytidylic acids and lipopolysaccharides in pregnant mice has produced pups with features of abnormal social behaviors, working memory deficit, and decrease in cognitive flexibility typically seen in human autism spectrum disorders. This alteration in offspring neuropsychological development is due to maternal immune activation, or mIA. High IL-6 and Th17 in these mothers may have caused intestinal microbiome changes that in turn alter the immune activation state, setting up a vicious cycle, eventually negatively affecting progenitor cells in the developing brain. Genome-wide association study of more than one thousand individuals with schizophrenia showed extremely significant link between the disease and two MHC class I alleles: HLA-B*08:01 and HLA-A*01:01 (71). In a more extensive follow up investigation published in 2014, the association of MHC with schizophrenia was indisputable. Among the background risks across all chromosomes, there was a single spike in significance to a *p* value of 10^−30^ for chromosome 6. One hypothesis is that with certain types of HLA, maternal immune activation when exposed to some pathogens can lead to exacerbated inflammatory response, which perturbs the neurogenesis in critical areas of the developing brain. Increase in the relative frequency of specific type of ILCs, perhaps ILC3, may be an intermediate step.

## Concluding Remarks

We review the ontogeny of immune defense in neonates and infants, a critical transition for the individual and sets up the conditions for health or disease in the future. The approach is heuristic, using the military as a paradigm, because of the many similarities between national defense and neonatal, infant immunity. Both must accept some while vigorously suppressing others. Detecting, discriminating, deploying, destructing, and de-escalating are what both military and immune systems do. The biotic process, competing for limited space and materials for survival is the purest form of warfare, utterly devoid of morality and morale. There are some obvious differences. Soldiers, sailors, and airmen are sentient beings—they can think and decide, while cells like macrophages, neutrophils, ILCs, or *E. coli* do not. At the cellular level, it is biochemistry, governed by affinity and reaction kinetics. However, the emergent properties are the same. Through their effector functions, ILCs may modulate the composition of the microbiota, controlling the initiation, progression, and regulation of immunoinflammatory responses in the host. The tasks, requirements, constraints, determinants, and trade-offs are the same. ILCs are type of immune effector cells, capable of a broad spectrum of functions and interactions within and/or with other groups of host cells. They play crucial roles in host defense, organogenesis, metabolic homeostasis, tissue remodeling, repair, and stem cell regeneration. Due to their appearance at early stages of ontogeny and their evolution throughout the embryonic development, ILCs can affect and maybe influenced by host cellular, molecular, and environmental factors, conferring the host a viable and robust source of plastic immune cells with capacity to adapt and contribute to the immune system from neonatal phase to the old ages of life.

Neonates, from the fetal stage, must build up a defense system that can go from quiescent to fully operational in the 5 min it takes to exit the womb either through the birth canal or *via* C-section, ending with the division of the umbilical cord, marking the beginning of independent living. It must accept all that is self and commensal while recognizing and attacking, repelling everything else, without prior knowledge of whether a given bacterium is commensal or pathogenic. Complicating the task, just as in the case of national security, this commensal or pathogenic status changes with time, dependent on the overarching context. In such CAS, studying the similar emergent properties permits and provides a different perspective; it allows us to answer what must be present given a particular set of demands under a given set of circumstances.

## Author Contributions

JY: concept, preparation, conceive, and writing of the manuscript HK: preparation and writing of the manuscript. AM: preparation and writing of the manuscript. BD: preparation 1100 and writing of the manuscript. ES: preparation and writing the manuscript. JB: preparation, conceive, and writing of the manuscript. VH: preparation, conceive, and writing of the manuscript. BB: concept, preparation, conceive, and writing of the manuscript.

## Conflict of Interest Statement

The authors declare that the research was conducted in the absence of any commercial or financial relationships that could be construed as a potential conflict of interest.
